# Radiological features of chronic pulmonary histoplasmosis: Easily mistaken for tuberculosis

**DOI:** 10.1371/journal.pntd.0013219

**Published:** 2025-08-08

**Authors:** David W. Denning, Adebimpe Onikan, Marcello Mihailenko Chaves Magri, Atisak Jiaranaikulwanich, Vitor Falcao de Oliveira

**Affiliations:** 1 Manchester Fungal Infection Group, Faculty of Biology, Medicine and Health, The University of Manchester, Manchester Academic Health Science Centre, Manchester, United Kingdom; 2 School of Medicine, The University of Manchester, Manchester, United Kingdom; 3 Department of Infectious Diseases, University of São Paulo, São Paulo, Brazil; 4 Department of Pathology, Faculty of Medicine, Ramathibodi Hospital, Mahidol University, Bangkok, Thailand; Erasmus Medical Center, Rotterdam University, NETHERLANDS, KINGDOM OF THE

## Abstract

*Histoplasma capsulatum* var *capsulatum* is an endemic respiratory pathogen presenting in various forms including miliary histoplasmosis, acute and chronic pulmonary histoplasmosis, and acute or subacute disseminated disease. The differential diagnosis of chronic pulmonary histoplasmosis (CPH) is broad, encompassing bacterial, fungal and malignant aetiologies. PubMed was searched for relevant articles on the radiological characteristics of CPH and the most common differential diagnoses of tuberculosis and chronic pulmonary aspergillosis. The Fleischner Society Glossary of Terms for Thoracic Imaging was used to analyze the features. The contribution of culture, antibody and antigen and PCR to the diagnosis of CPH is summarized. Cavitation and pulmonary nodules are the most common features of CPH. Pleural effusion, pleural thickening, intrathoracic lymphadenopathy and bronchiectasis are not characteristic of CPH; uncommonly CPH can be complicated by an aspergilloma. Data on the radiologic features of CPH are derived primarily from the USA, Brazil, and China. CPH can be diagnosed by respiratory fungal culture (using extended culture times) or *Histoplasma* PCR (although data are scarce) and serum *Histoplasma* antigen and antibody. Data on bronchoscopy sampling for antigen are lacking. In patients with pulmonary cavitation without a confirmed diagnosis of tuberculosis or aspergillosis should be evaluated for CPH.

## 1. Introduction

Histoplasmosis covers various clinical and radiologic presentations that occur following inhalation of the fungus *Histoplasma capsulatum var. capsulatum.* This species has recently been split into several other clades and genotypes including probable new species such as *Histoplasma ohiense*; here all referred to as simply *H. capsulatum. H. capsulatum* is endemic in NE India, the eastern states of the US, Central America, South America and other parts of Southeast Asia, [1] Europe, [2] and Africa [3–5]. *H. capsulatum* is acquired from the environment except in rare cases of transmission via organ transplantation [6]. *H. capsulatum* fungus exhibits thermal dimorphism. It is found as a mould in the environment but at 37°C it exists as a yeast [7]. The inhaled microconidia transform into pathogenic yeast either within phagocytes (macrophages) or extracellularly, and often remain latent for years [7].

The presentation of pulmonary histoplasmosis varies from asymptomatic to severe and fatal forms. Most often the fungus is acquired asymptomatically in an individual and is usually eliminated quickly [8,9], sometimes leaving only randomly distributed small calcified lesions on a chest X-ray [10,11]. The three main pulmonary manifestations of histoplasmosis are acute pulmonary histoplasmosis, chronic pulmonary histoplasmosis (CPH), including *Histoplasma* nodules, and progressive disseminated histoplasmosis in the immunocompromised host [12]. Miliary histoplasmosis is described, but is rare in acute pulmonary histoplasmosis, and slightly more common in AIDS and other highly immunocompromised states. Dissemination is well described; to the gastrointestinal tract or adrenals or occasionally to the larynx, heart valve, brain, mediastinal structures, retinae or other organs, even in non-immunocompromised people [10,13,14]. Here the focus is CPH.

CPH has a time course of weeks to months. Classically CPH was defined by the presence of cavitary lung disease (chronic cavitary pulmonary histoplasmosis (CCPH) [15–17]. However, CPH in modern literature is defined more broadly by the chronicity of symptoms with many different radiographic features [15,17]. Clinically, it presents with respiratory symptoms of chronic cough without hemoptysis, dyspnea as well as constitutional symptoms of weight loss and fever [16,18–20].

CPH may develop after acute pulmonary histoplasmosis with a natural course of cavitation, fibrosis and progressive pulmonary insufficiency [16,18,20]. In other cases, the initial onset may not have been recognized as histoplasmosis and the patient may present later in life with an incidental finding of an asymptomatic pulmonary nodule found on a chest radiograph [21].

CPH is more common in the middle decades of life, male sex in the older literature, white race, and linked to some immunosuppression or chronic lung disease [15,20]. Cavitary disease is usually associated with the presence of structural lung diseases such as emphysema or chronic obstructive pulmonary disease (COPD). Patients may develop CPH without COPD or a smoking history, particularly women [15].

CPH is estimated to affect no more than 1 in 2000 of those exposed and infected [22]. Following a large outbreak in Indianapolis in 1978, 62 of 741 (8.4%) symptomatic individuals developed cavitation – some acute, some chronic [20]. Previous reviews of this topic with detailed radiological descriptions were in 1976, 1996, and 2006 [22–24], and broader reviews in 2004, 2020, 2021, and 2023 [9,10,17,25]. This review seeks to provide an overview of the characteristics of CPH, with a focus on radiological aspects, and summarizes how the diagnosis may be made.

## 2. Methods

The radiological features of CPH were sought by searching the PubMed database on July 10th 2024, for relevant primary papers and reviews on radiological characteristics dating back to 1964, with search terms including “chronic pulmonary histoplasmosis”, “Histoplasma”, “cavitary”, “chronic cavitary pulmonary histoplasmosis”, “diagnosis”, “radiography”, and “tomography”. Older papers were then retrieved from the reference lists. Highly reported features were selected and described in detail. The imaging features were analyzed according to the Fleischner Society Glossary of Terms to ensure standardized terminology [26].

CPH was defined by a duration of illness of at least 6 weeks, with confirmatory findings of a positive respiratory culture of *H. capsulatum* or in a few instances, microscopic or histologic evidence of small intracellular yeasts, a positive *Histoplasma* antibody or antigen assay in urine and serum. This excluded the work of Goodwin describing early features of CPH by sequential chest X-rays as these patients had a subacute presentation, which usually resolved [27]. Some elements of the large series of Wheat and colleagues [20] are included as patients with acute and chronic presentations of cavitary histoplasmosis. A narrative review methodology was adopted to summarize current knowledge in the field and identify gaps for future research.

## 3. Results

We identified seven primary papers and several reviews describing the radiological features of CPH. We specifically analyzed key retrospective case series [15,20,28–32] that detail the radiological features of CPH, all from the USA, Brazil, or China. The common and uncommon findings were emphasized in various reviews [15,23,33–35]. Cases were documented in adults ranging from their 20s to their 70s, with the duration of disease before presentation (based on symptoms) was months or years [28,36].

### 3.1. Epidemiology

Our understanding of CPH has changed in recent years, including our understanding of the epidemiology, clinical and radiological manifestations [15,37]. The changing epidemiology of histoplasmosis and the increasing mortality and morbidity due to the increasing numbers of people living with HIV and other more subtle immunosuppressed states make the recognition and diagnosis of histoplasmosis even more essential [38,39].

Histoplasmosis exhibits a far broader geographic distribution than previously recognized, with endemic foci confirmed across the Americas, Africa, Asia, and parts of Oceania and Europe [40]. However, CPH is considered an underdiagnosed entity, and its true incidence and prevalence remain poorly defined [3,41]. In historical data from the US, CPH was identified in 7.2% of patients admitted to a tuberculosis sanatorium, reflecting its frequent misdiagnosis as smear-negative pulmonary tuberculosis in endemic areas [42].

Mortality data specific to CPH are also scarce. However, in a cohort of 90 patients with CCPH followed for a mean of 40 months, 10% died from histoplasmosis-related pulmonary disease, and an additional four patients died following surgery [19]. Given the limited awareness and diagnostic capacity, particularly in low-resource settings, CPH is likely underdiagnosed and often misclassified as tuberculosis, especially in regions where both diseases are endemic, such as Africa and Southeast Asia.

As *H. capsulatum* lives in soil, activities that result in the disruption of soil (farming, gardening, renovation and construction work) can cause aerosolization and subsequent inhalation of the fungus microconidia. Bird and bat excretions act as fertilizers, enriching the soil with nitrogen and making soil more suitable for *H. capsulatum* growth [9]. Bats may also be infected by the fungus [43]. Therefore, activities such as the exploration of bat caves (spelunking) or cleaning of chicken coops have been linked to acute pulmonary histoplasmosis, but not CPH directly [1,44].

### 3.2. Clinical presentation

CPH typically presents with chronic cough and marked weight loss, often accompanied by fatigue, fever, and dyspnea. Although hemoptysis is not a predominant feature, it may be observed in later stages, particularly in association with cavitary lesions, and, when substantial, may indicate a superimposed aspergilloma [16–18].

Symptoms vary according to disease stage. In a series of 228 CCPH cases, early disease was characterized by systemic symptoms such as fever (42%) and chest pain (35%), whereas in late-stage disease, pulmonary symptoms predominated, including cough (76%), sputum production (61%) and hemoptysis (36%). Weakness (35%) and weight loss (60–64%) were consistently reported, regardless of disease stage, underscoring the chronic inflammatory burden [17]. These data suggest a shift from constitutional to pulmonary symptoms as the disease progresses, highlighting the importance of early recognition to prevent cavitation and pulmonary destruction.

This profile of symptoms closely resembles that of pulmonary tuberculosis, albeit often with less severity. Notably, chest pain in CCPH tends to be a deep, aching discomfort, differing from the pleuritic pain seen in tuberculosis. Chronic pulmonary aspergillosis (CPA) remains a key differential diagnosis in patients with similar clinical and radiological profiles.

### 3.3. Diagnosis

Pulmonary histoplasmosis, and especially CPH, is largely underdiagnosed in many regions of the world [10,39], partly because of the difficulty of diagnosis, as prolonged fungal culture and antibody serology are unavailable in many locations [10]. Inconsequence, diagnosis of pulmonary histoplasmosis relies on laboratories with specific skills and trained personnel [6]. As a result, diagnostic delays and missed cases are frequent [6].

Another risk factor for delay in diagnosis of CPH is having a comorbid pulmonary disease [6,17,25]. Without awareness of CPH, it can be mistaken for an exacerbation of an existing lung condition, malignancy or other infectious diseases. Reactivation pulmonary tuberculosis and CPA are two important differential diagnoses for CPH [45,46]. CPH and other forms of histoplasmosis are often mistaken for other diagnoses. This is especially true in non-endemic regions where there may be a lack of clinical suspicion [47]. Unnecessary invasive testing, such as lung biopsies, may be carried out [21,48–50]. Patients may undergo the wrong treatment [38,39].

The diagnosis of CPH is based on laboratory methods of respiratory fungal culture, antibody testing, antigen testing, and occasionally histopathology [9,17,25]. Histologically, *H. capsulatum* in tissue samples is a small intracellular yeast and can be a fast method of diagnosis, but is rarely done in CPH. Clinical and radiological manifestations of the disease are nonspecific; the radiological manifestations of CCPH overlap with tuberculosis, non-tuberculous mycobacterial infection, and CPA especially.

Probably the most sensitive diagnostic tests for CCPH are respiratory fungal culture and anti-*Histoplasma* IgG antibody. *H. capsulatum* can take anywhere from 2 weeks to 8 weeks for a colony to grow [51–53]. Cultures should be maintained at 35–37°C to maintain the yeast phase which poses no threat to laboratory technicians, especially in non-endemic regions where the general population hasn’t been exposed to the fungus [54]. Conversion from the yeast phase to mycelial phase by culture at 20–25°C requires handling in a Category 3 laboratory [53]. Staining of the mycelia with lactophenol cotton blue will show tuberculate macroconidia characteristic of the *H. capsulatum* mould but also manifest in other species of fungi such as the *Sepedonium* species [53]. Misidentification may cause false positives for histoplasmosis, underscoring the importance of specific testing to ensure diagnostic accuracy.

Recent advancements in diagnostics aim to differentiate histoplasmosis from tuberculosis by focusing on more sensitive and specific tests, particularly antigen detection methods. Antigen detection plays a pivotal role in diagnosing pulmonary histoplasmosis, particularly in acute forms and in disseminated disease among immunosuppressed patients or those with severe illness [55,56]. In contrast, sensitivity is significantly lower in subacute and CPH due to a reduced fungal burden. In contrast, urine and serum antigen detections yielded positive results in only one out of four (25%) tested patients, while four out of five (80%) patients tested positive for *Histoplasma* serology [57]. In a multicenter study, antigen was detected in 30% of subacute cases and 88% of chronic pulmonary cases [56], although the latter group had a small sample size. In a larger study from Venezuela involving 251 patients with CPH, urinary antigen positivity was low (48%) [58].

The detection of antigens in bronchoalveolar lavage (BAL) demonstrates 93.5% sensitivity for pulmonary histoplasmosis [59]. However, the majority of patients had progressive disseminated histoplasmosis, with only five cases characterized by cavitary histoplasmosis [58]. The sensitivity of BAL antigen detection in these patients reached 100% following sample pretreatment with heat and EDTA. Therefore, while antigen testing alone is insufficient to rule out CPH, it remains a valuable adjunct when integrated with serologic, radiographic, and microbiologic findings, especially in patients for whom invasive sampling is not feasible [60].

An alternative to culture would be specific PCR on respiratory samples, but there is no commercialized system, and data are scanty. PCR on BAL and bronchial fluid probably have high sensitivity and specificity compared to traditional culture [61–65], but no formal analytical diagnostic studies for CPH are published. Furthermore, these studies did not address any specific clinical syndrome of histoplasmosis including CPH. Using ITS primers and sequencing on sputum PCR in patients with possible pulmonary tuberculosis in Nigeria, 18 of 213 (8.5%) were positive for *H. capsulatum* but no radiological or clinical course data is provided to assess if the diagnosis was CPH, or colonization or another form of histoplasmosis [64].

The initial antibody assays were immunodiffusion, detecting IgG and IgM precipitating antibodies to H and M bands or complement fixation to *Histoplasma* yeast antigens [16,20,66]. This assay is the only commercially available format and test available globally [67,68]. There is no formal performance data of these assays published in CPH. Older studies of CPH patients found approximately 75% of patients had a positive immunodiffusion assay and >90% had either a positive CF, radioimmunoassay or EIA test, with good specificity is even in persons from a *Histoplasma* endemic area [53,66]. A Western Blot antibody assay performs well and is available in a reference laboratory in Brazil [69].

### 3.4. Radiological findings

The radiological manifestations of pulmonary histoplasmosis are nonspecific and taken in isolation the differential diagnoses are broad and include infectious aetiologies and malignancy [48]. Table 1 summarizes the radiological findings of different forms of pulmonary histoplasmosis.

**Table 1:** Radiological features of different forms of pulmonary histoplasmosis.

	Typical radiological manifestations	Commentaries
**Acute pulmonary histoplasmosis**	Areas of consolidation, pulmonary nodules (solitary or multiple), and mediastinal/hilar lymphadenopathy.	Rarely miliary pattern and pericardial/pleural effusion.
**Chronic cavitary pulmonary histoplasmosis**	Chronic consolidation with progressive cavitation and volume loss, usually in the upper lobes.	Predominantly seen in patients with emphysema.
**Chronic histoplasmosis without cavitation**	Calcified pulmonary nodules, calcified mediastinal/hilar nodes, bronchiolitis, histoplasma, and fibrosing mediastinitis.	Nodules were the most common feature of chronic pulmonary histoplasmosis.
**Disseminated histoplasmosis**	Diffuse micronodular or air-space opacities.	Miliary pattern may also be observed.
**Miliary histoplasmosis**	Micronodules with a random and diffuse distribution throughout the lungs	Miliary nodules have wide differentials; culture, antigen, and histopathology are key, especially in immunocompromised patients.

Chest radiography is the most common imaging modality used for pulmonary disease. Yet its utility in delineating specific radiologic features of pulmonary histoplasmosis from other presentations is underexplored. Thoracic imaging has improved in recent decades and, in occasional cases where the radiograph appears normal, CT may discover subtle signs. In the 2006, series by Kennedy and colleagues of 46 patients diagnosed with CPH in a tertiary practice, chest X-rays revealed 74% of patients with nodules but CT scan revealed a further 93% with pulmonary nodules [15]. However, even with the use of chest CT, histoplasmosis can be indistinguishable from other infectious diseases. It is important to consider the epidemiological history, such as travel to histoplasmosis-endemic areas or participation in activities that pose a risk of exposure to *Histoplasma*. Additionally, diagnostic tests for histoplasmosis should be performed when the radiological findings are compatible, especially if tests for tuberculosis are negative. However, many countries lack adequate diagnostic tools to provide rapid and accurate results for histoplasmosis. Ideally, improved funding and greater awareness of this mycosis in many regions would help prevent mortality [68].

#### 3.4.1. Primary infection and the Ghon focus.

Following inhalation of *H. capsulatum*, a primary infection occurs, similar to tuberculosis. First properly described in 1955 [70], the characteristic features are an evolving combination of lung and mediastinal lymphadenopathy over a period of weeks. Initial areas affected with soft areas of consolidation or large nodular infiltrates then develop caseous necrosis with fibrotic healing, and then later the development of calcification. This calcific response is usually more pronounced than that of tuberculosis; parenchymal calcifications >4 mm and hilar or mediastinal nodal calcifications >1 cm are 80% more often seen in histoplasmosis than tuberculosis, based on autopsy examination. Rarely are the *Histoplasma* organisms completely killed, so long term latency should be assumed.

Large exposures to *H. capsulatum* may lead to acute pulmonary histoplasmosis with severity of symptoms corresponding to the inoculum size. Numerous point source outbreaks are described with the most severe recent one being in the Dominican republic, which left 3 of 32 tunnel workers (10%) dead, among 28 (93%) who were hospitalized, nine (30%) in intensive care [71].

#### 3.4.2. Pulmonary cavitation.

Cavitary disease, which is a hallmark feature of reactivation tuberculosis, may also manifest in CPH [72]. Cavitary lesions associated with histoplasmosis may manifest with thin or thick walls depending on the stage of development [16,17]. Cavitary disease may be unilateral or bilateral, and minor in extent, or far advanced [30]. Cavities may also contain a fungus ball [17,31,73], probably attributable to *Aspergillus* and therefore a co-infection (see below). There may be associated pleural thickening adjacent to the cavity [33].

CCPH is defined by chronic consolidation with associated cavitation [16,17,74]. Older literature indicated that the apical posterior segments of the upper lobes were most frequently affected, as in tuberculosis [20,75]. However, recent descriptions and images show isolated cavities in other lung lobes. CCPH left untreated usually progresses and leads to progressive pulmonary insufficiency and there is a small risk of disseminated disease [10,17,19,30,76]. The cavitation may grow in size and is then described as a ‘marching cavity’, to involve an entire lobe, eventually resulting in volume loss [17,18,36]. It is hypothesized that ‘marching cavities’ only occur in people residing in endemic regions with continuing exposure to airborne *Histoplasma* spp., but this is uncertain, partly because the radiological criteria for this term remain vague and appearances are heterogeneous.

**Fig 1:**
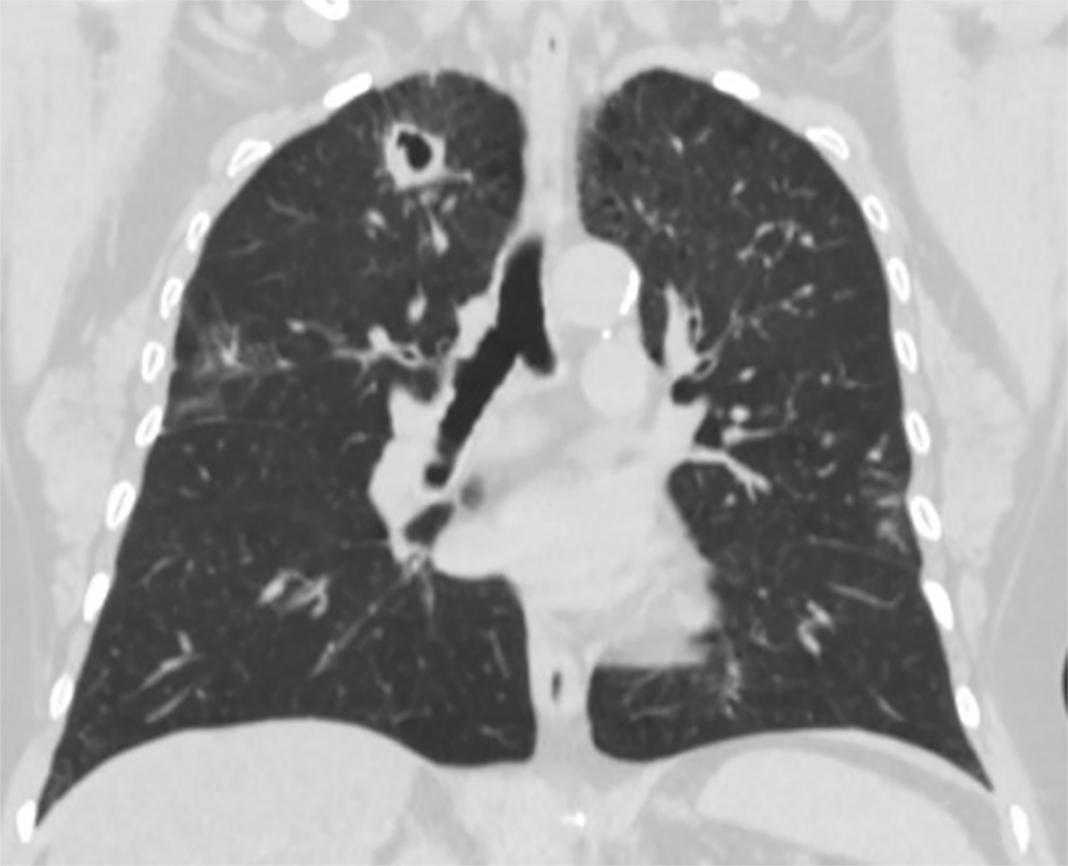
A 62-year-old woman with a history of COPD, asthma, and type 2 diabetes presented with chronic dyspnoea. A chest CT revealed a pulmonary cavitation in the right upper lobe. Bronchoalveolar lavage culture grew *Histoplasma capsulatum*.

Sometimes the visible lesion is a mass-like lesion with very little cavitation. Cavitary disease occurs opportunistically mostly in those with previous lung disease, without immunocompromise [16]. There are important links between smoking and the development of cavitary disease, probably a function of emphysema, but cases also arise in the under 40s [15]. The disease usually progresses without treatment, leading to pulmonary insufficiency [18,19,30]. Collapse of the cavity and progressive pleural thickening with pulmonary fibrosis may also result in volume loss and eventually in a destroyed lung, akin to chronic fibrosing pulmonary aspergillosis [77]. Cavitary disease may also heal to form a residual scar or remain stable for years (Fig 1) [76,78].

#### 3.4.3. Dual infection with *Histoplasma* and *Aspergillus.*

A fungal ball (aspergilloma) is characteristic of CPA, including simple aspergilloma, but it may also occasionally be seen in CPH, reflecting a dual infection with CPA and CPH [22,73,79,80]. Marked hemoptysis is one clue to superinfection of CPH with *Aspergillus*. Although an aspergilloma is a common form of secondary *Aspergillus* infection in lung cavities, cavity wall and/or pleural thickening can also occur in the absence of a fungus ball [78].

The real frequency of dual infection remains understudied, with few reports in the literature. This is partly due to overlapping radiological features, the low sensitivity of respiratory fungal cultures for *Aspergillus* spp. and *H. capsulatum*, and inadequate documentation by IgG antibody testing. A recent small study of multidrug-resistant tuberculosis cases in a *Histoplasma* endemic area in Indonesia found 14 (28%) and 16 (32%) of 50 patients to have positive *Histoplasma* or *Aspergillus* IgG, respectively, with 6 (12%) to have both IgG antibodies present [81].

#### 3.4.4. Pulmonary nodules.

A pulmonary nodule is defined as a ‘circumscribed, typically rounded opacity’ less than or equal to 3 cm in diameter [26]. The features of the nodules vary and are nonspecific [33]. Pulmonary nodules are best investigated with CT and are an important differential diagnosis of early carcinoma of the lung and nodules attributable to *Aspergillus*, *Coccidioides* or non-tuberculous mycobacterial (NTM) infection to name a few differential diagnoses [82]. Common to pulmonary tuberculosis, NTM lung disease, CPA and CPH are manifest as both cavitary lesions and round nodular lesions [83,84]. Additionally, these nodules may present asymptomatically years after an acute infection and the epidemiological exposure may be obscure [2] or they may also present in association with chronic low-grade symptoms.

**Fig 2:**
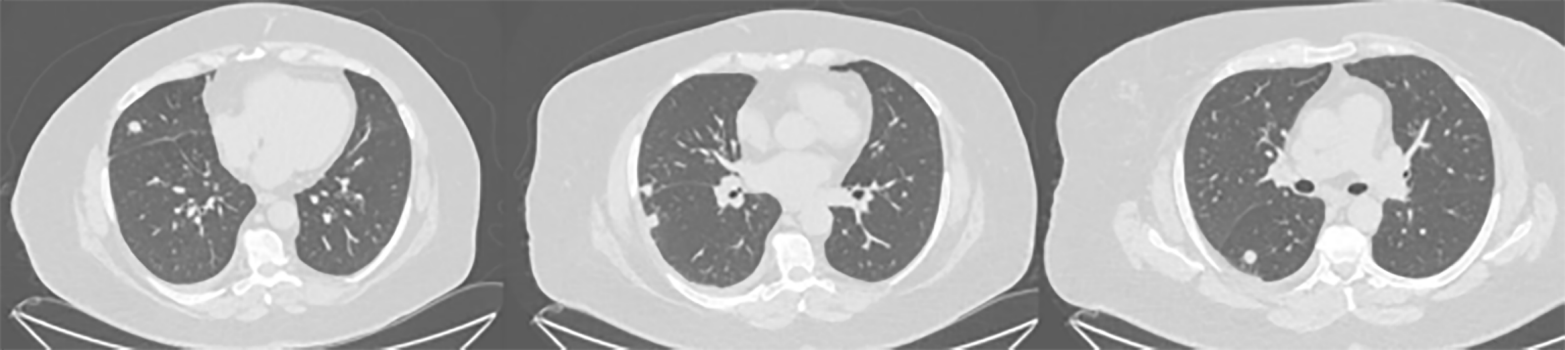
A 63-year-old woman with hypertension, obesity, and a history of smoking. A chest CT showed multiple nodules in the lungs. The patient was diagnosed with pulmonary histoplasmosis after the biopsy and positive serology.

**Fig 3:**
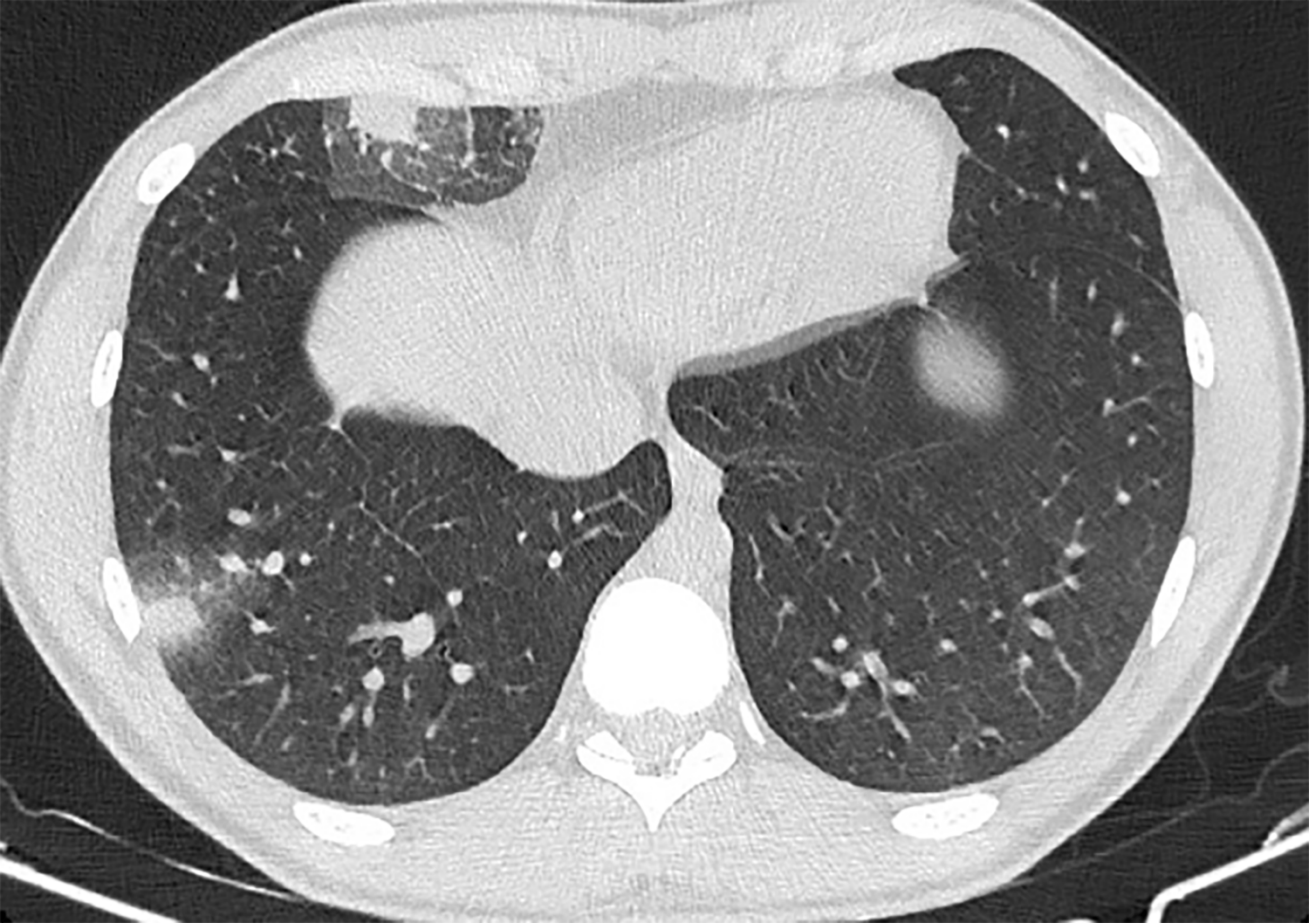
A 24-year-old man with a history of Crohn’s disease, currently on infliximab and azathioprine, presented with a two-week history of cough, fever, chills, night sweats, and dyspnea after visiting a cave. A chest CT revealed two nodules with slightly irregular contours and a ground-glass halo, located in the peripheral region of the right lower and middle lobes. He was diagnosed with probable pulmonary histoplasmosis after positive serology.

Nodules associated with histoplasmosis may be solitary or multiple (Fig 2) [35], and typically have a lower lobe predominance and are usually peripherally located, adjacent to the pleura [33]. Nodules may present with an associated ground-glass halo sign (Fig 3) [35], and may also demonstrate cavitation [35] or undergo necrosis. These lesions consist of inflammatory cells recruited as part of the immune response; a ‘residual granuloma’ surrounded by the normally aerated lung. Solitary pulmonary nodules or masses are often investigated as suspected malignancies [21,49,50] although the occurrence of *Histoplasma* nodules is probably rare [21,41]. FDG-PET has a high sensitivity but low specificity to delineate malignancy from infectious aetiologies of such pulmonary nodules [49].

*Histoplasma* nodules are typically rounded opacities presenting with both well-defined or ill-defined margins and with regular or spiculated borders. A dominant nodule may be associated with smaller satellite nodules or with broncho-vascular beading [35]. Nodules may progressively grow in size as a result of an abnormal host response. Fibrosis at the periphery of the nodule leads to growth at an average rate of 1.7 mm per year [35]. These nodules may be non-calcified or contain central/laminar/diffuse calcification. Those with central calcification are often called a histoplasmoma and resemble a “target lesion” [35]; they may grow up to 4 cm in diameter. Calcification in a nodule is a clue to histoplasmosis, rarely seen in malignancy.

Notably, nodules in the lungs associated with histoplasmosis may present with concomitant extrapulmonary nodules, such as in the liver and the spleen, which are visible on plain radiography, if calcified [35].

#### 3.4.5. Micronodules and miliary histoplasmosis.

**Fig 4:**
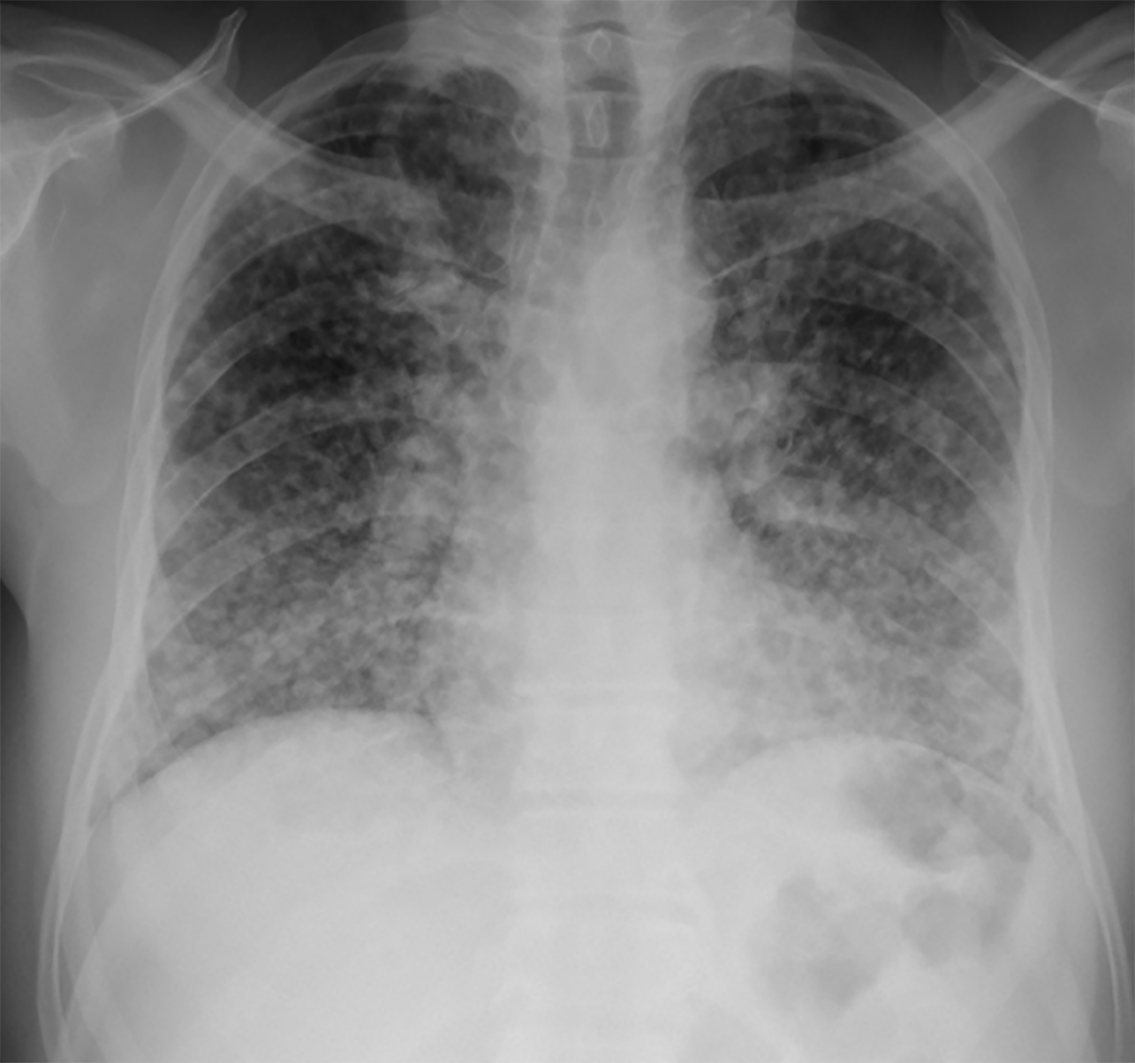
A 55-year-old man with no previous comorbidities presented with a 3-week history of cough, expectoration, fatigue, fever, night sweats, dyspnoea, and weight loss. A chest X-ray showed a miliary pattern. The patient was diagnosed with histoplasmosis after lung biopsy and positive serology.

A micronodule is defined as a circumscribed lesion less than 6 mm in diameter [26]. In acute disseminated histoplasmosis micronodules may appear diffusely distributed throughout the lungs, generally described as a miliary pattern; multiple small (<3 mm) pulmonary nodules of similar size that are randomly distributed throughout both lungs. Hematogenous spread of the fungus from re-infection from a very recent (usually <3 days) substantial exposure results in multiple foci of inflammation distributed throughout the lung, without lymphadenopathy or pleural effusion [35]. The differential diagnosis is wide, including tuberculosis, aspergillosis and coccidioidomycosis. Spontaneous resolution over several weeks or gradual deterioration and death may follow, hence antifungal treatment is usually given (Fig 4).

#### 3.4.6. Thoracic lymphadenopathy.

Hilar and mediastinal lymphadenopathy in histoplasmosis is a feature of a primary infection (see above), and may persist. Lymphadenopathy is often concomitant with features of consolidation, pulmonary nodules and/or histoplasmoma, but is rarely seen in those with cavitary disease [20]. Over time lymph nodes may calcify.

Lymphadenopathy is rare in CPA, NTM and in the cavitary forms of CPH and finding thoracic lymphadenopathy in association with cavitary disease should alert the clinician to other diagnoses (tuberculosis, lymphoma, other malignancy, etc).

#### 3.4.7. Calcified lesions.

Calcified lesions include calcified pulmonary nodules, calcified lymph nodes and broncholiths [18,85,86]. The pattern of calcification of the nodules may be central, diffuse or laminar and is seen on CT [35]. There may be multiple calcified nodules. As the infection heals, calcification occurs as a result of the deposition of calcium hydroxyapatite by necrotic cells within the nodule. A characteristic feature of resolved primary infection (perhaps most commonly seen in children) is paratracheal lymphadenopathy with calcification, sometimes called ‘mulberry calcification’ because of its multilobed appearance. Calcified lymph nodes can lead to a complication known as a broncholith. This manifests as erosion of a node into an airway obstructing the airway [35,86]. The complications vary and include atelectasis – partial/total collapse of the lung distal to the obstruction. This may be visualized on chest X-rays. Calcified nodules are much more likely to be benign and point towards an infectious etiology.

#### 3.4.8. Pleural effusions.

**Fig 5:**
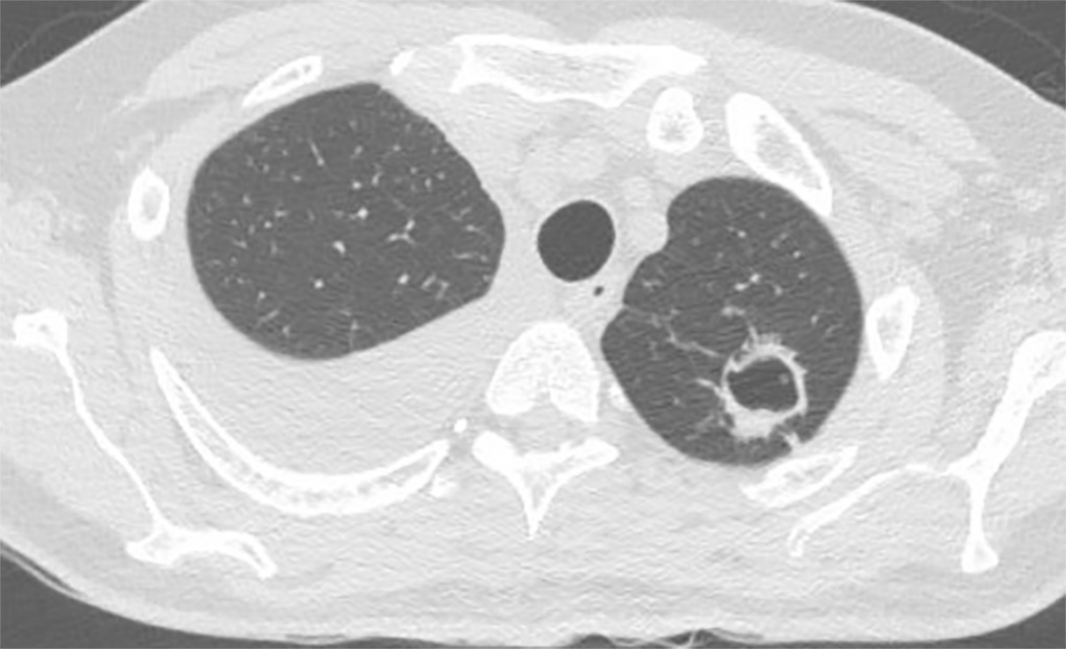
A 48-year-old man with dermatomyositis, currently undergoing combination therapy with leflunomide and methotrexate, presented with chronic dyspnoea. A chest CT scan revealed a right chronic pleural effusion (a rare finding in CPH) with associated thickened pleura and a cavitary lesion in the left upper lobe. He was diagnosed with pulmonary histoplasmosis following positive serology with high titers and confirmation via lung biopsy.

Pleural effusions are rare in all forms of histoplasmosis [16,19,20] (Fig 5). Fluid may appear in a cavity, or emphysematous bullae adjacent to an area of histoplasmosis, usually for a short period, before that area consolidates or fibrosis. Pleural effusion may be seen in pulmonary tuberculosis, but is rare in NTM lung disease, CPA and CPH.

Pleural thickening is highly suspicious for CPA, but seen in the acute phase of pulmonary tuberculosis (usually resolving with therapy) but appears to be uncommon in CPH. Pleural thickening in CPA can be seen in association with cavities (‘para-cavitary’ thickening), nodules or aspergilloma fungal balls [45].

#### 3.4.9. Bronchiectasis and broncholithiasis.

Bronchiectasis is not associated with histoplasmosis, unless there is another reason for it. In contrast, histoplasmosis is one of the commoner causes of broncholithiasis, perhaps the most common cause in endemic areas for histoplasmosis, where tuberculosis is less frequent [85,86]. Calcification of lymph nodes precedes broncholithiasis by several months or years although the time frame is shorter in children.

#### 3.4.10. Fibrosing mediastinitis.

**Fig 6:**
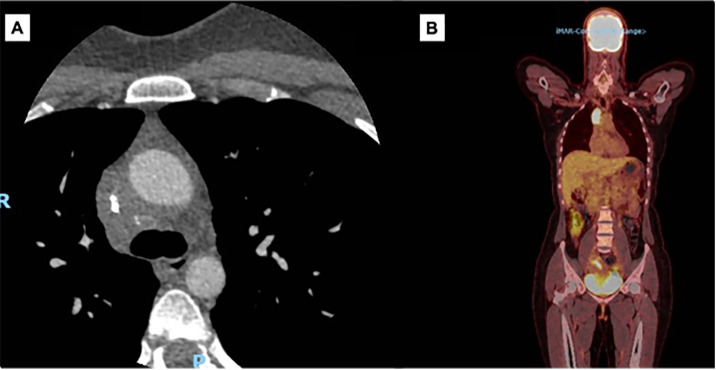
A. A 31-year-old woman with no comorbidities presented with facial plethora and oedema of the cervical region and upper limbs. A CT angiography scan revealed a lesion measuring 5.4 × 3.0 × 4.4 cm, with approximately 75% reduction in the caliber of the superior vena cava. She was diagnosed with pulmonary histoplasmosis after positive serology results obtained by two different methods. The mediastinal biopsy revealed only fibrosis. B. PET/CT scan showed a mediastinal mass with high glycolytic activity.

Fibrosing mediastinitis is a serious presentation associated with pulmonary histoplasmosis [16,18,35,87]. It is visualized as the accumulation of fibrotic tissue in the mediastinum structures, especially the middle mediastinum (Fig 6). This can lead to compression of various mediastinal structures such as the superior vena cava [31]. Fibrosing mediastinitis caused by *H. capsulatum* is the most common benign cause of superior vena cava syndrome [35]. The chest X-ray usually shows mediastinal widening [18,35,88].

Among infections such as CPA, only CPH is known to cause mediastinal fibrosis. In patients with fibrosing mediastinitis, attribution to histoplasmosis can be difficult. The differential diagnosis includes lymphoma, tuberculosis, aspergillosis (possibly linked to chronic granulomatous disease), idiopathic hyalinizing fibrosclerosis, esophageal perforation, sarcoidosis, silicosis or other biomass exposures, immunoglobulin G4 (IgG4)-related disease, prior mediastinal radiation or anti-programmed death (PD)-1 therapy (pembrolizumab) or autoimmune diseases [53,54].

### 3.5. Treatment

While not every patient with CPH requires treatment, [89] most do. Active therapeutics include itraconazole and liposomal amphotericin B. The antifungal therapy halts disease progression, facilitates the clearance of *H. capsulatum* from sputum, and leads to the resolution of pulmonary infiltrates in approximately two-thirds of patients [90].

Notably, an important interaction exists between the first-line antimycobacterial drug rifampicin and the triazole antifungal agents. Rifampicin acts as an enzyme inducer. Induction of the cytochrome 3A4 enzyme causes a reduction in serum concentrations of itraconazole [91,92]. This highlights the necessity of accurate identification of the causative agent as treatment pathways are widely different for reactivation of tuberculosis and CPH and co-administration of many antimycobacterial agents and triazole antifungal medication is contraindicated [93].

Outcomes in the case of CPH are affected by whether or not treatment is given and also the length of time between diagnosis and treatment [30]. By reducing functional capacity, CPH impairs work capacity and quality of life for several months [94]. In some cases of persistent cavitation, surgery may be indicated [16,75]. Corticosteroids and surgical resection have been the mainstays of therapy for fibrosing mediastinitis, with limited success. Recently rituximab has been used with approximately 50% responding over 24 months [95].

## 4. Limitations

A significant limitation to the description of the radiographic features of CPH is the small datasets in the recent literature. Rubin in 1959 reported on 78 patients [19] and Parker in 1970 on 408 patients [30]. Among more recent series, with CT scanning, the largest dataset for CPH was 46 patients in 2007 [15]. Recent changes in the definition of CPH meant that older studies which largely focused on patients with cavitary disease were excluded. Another limitation is the predominant use of chest X-rays rather than CT.

A study of the radiological features of these conditions could ideally be carried out with a large database of radiological reports for comparison and analysis, preferably several hundred cases. Another limitation was inconsistent terminology used in the pertinent literature. The Fleischner Society Glossary of Terms has provided a useful means for analysis and comparison of features as detailed in the literature. An intrinsic problem to this disease area is co-infection, of CPA and aspergilloma, of cavitary pulmonary tuberculosis and CPA, and other infections such as non-tuberculous mycobacterial infection. In many instances, these conditions were not ruled out in assessing the radiological findings. In addition, both CPH and CPA tend to complicate other pulmonary conditions, so separating out the fungal disease from the underlying chest condition can be problematic.

## 5. Conclusion

We review the radiological features of CPH, with a focus on the chronic cavitary form. Since the radiological findings of CPH are nonspecific, the diagnosis is often mistaken for tuberculosis. CPH presents a wide spectrum of thoracic manifestations principally marked by cavitation and calcified pulmonary nodules. Lymphadenopathy is common, except in those with chronic cavitary disease. Co-infection with *Aspergillus* is probably underappreciated. This study helps to better characterize the radiological profile of CPH, potentially reducing diagnostic delays and missed cases.

## Abbreviations

**:**
BAL: bronchoalveolar lavage
COPD: chronic obstructive pulmonary disease
CPA: chronic pulmonary aspergillosis
CPH: chronic pulmonary histoplasmosis
CCPH: chronic cavitary pulmonary histoplasmosis
NTM: non-tuberculous mycobacterial

## References

[1] Lottenberg R, Waldman RH, Ajello L, Hoff GL, Bigler W, Zellner SR. Pulmonary histoplasmosis associated with exploration of a bat cave. Am J Epidemiol. 1979;110(2):156–61. doi: 10.1093/oxfordjournals.aje.a112800 572635

[2] Antinori S, Giacomelli A, Corbellino M, Torre A, Schiuma M, Casalini G, et al. Histoplasmosis diagnosed in Europe and Israel: a case report and systematic review of the literature from 2005 to 2020. J Fungi (Basel). 2021;7(6). 10.3390/jof7060481 PMC8231918 34198597

[3] Bongomin F, Kibone W, Atulinda L, Morgan B, Ocansey B, Storer ISR, et al. Frequency of fungal pathogens in autopsy studies of people who died with HIV in Africa: a scoping review. Clin Microbiol Infect. 2024;30(5):592–600. doi: 10.1016/j.cmi.2023.12.016 38145865 PMC11103628

[4] Antinori S. *Histoplasma capsulatum*: more widespread than previously thought. Am J Trop Med Hyg. 2014;90(6):982–3. doi: 10.4269/ajtmh.14-0175 24778192 PMC4047757

[5] Azar MM, Loyd JL, Relich RF, Wheat LJ, Hage CA. Current concepts in the epidemiology, diagnosis, and management of histoplasmosis syndromes. Semin Respir Crit Care Med. 2020;41(1):13–30. doi: 10.1055/s-0039-1698429 32000281

[6] Buitrago MJ, Martín-Gómez MT. Timely diagnosis of histoplasmosis in non-endemic countries: a laboratory challenge. Front Microbiol. 2020;11:467. doi: 10.3389/fmicb.2020.00467 32269555 PMC7109444

[7] Mittal J, Ponce MG, Gendlina I, Nosanchuk JD. *Histoplasma capsulatum*: mechanisms for pathogenesis. Curr Top Microbiol Immunol. 2019;422:157–91. doi: 10.1007/82_2018_114 30043340 PMC7212190

[8] Barros N, Wheat JL, Hage C. Pulmonary histoplasmosis: a clinical update. J Fungi (Basel). 2023;9(2). 10.3390/jof9020236 PMC9964986 36836350

[9] Kandi V, Vaish R, Palange P, Bhoomagiri MR. Chronic pulmonary histoplasmosis and its clinical significance: an under-reported systemic fungal disease. Cureus. 2016;8(8):e751. doi: 10.7759/cureus.751 27688988 PMC5037059

[10] Wheat LJ, Conces D, Allen SD, Blue-Hnidy D, Loyd J. Pulmonary histoplasmosis syndromes: recognition, diagnosis, and management. Semin Respir Crit Care Med. 2004;25(2):129–44. doi: 10.1055/s-2004-824898 16088457

[11] Richmond BW, Worrell JA, Bastarache JA, Gervich DH, Slattery WR, Loyd JE. Histoplasmomas of uncommon size. Chest. 2013;143(6):1795–8. doi: 10.1378/chest.12-2071 23732591

[12] Marukutira T, Huprikar S, Azie N, Quan S-P, Meier-Kriesche H-U, Horn DL. Clinical characteristics and outcomes in 303 HIV-infected patients with invasive fungal infections: data from the Prospective Antifungal Therapy Alliance registry, a multicenter, observational study. HIV AIDS (Auckl). 2014;6:39–47. doi: 10.2147/HIV.S53910 24648769 PMC3958502

[13] Franklin AD, Larson L, Rauseo AM, Rutjanawech S, Hendrix MJ, Powderly WG, et al. A comparison of presentations and outcomes of histoplasmosis across patients with varying immune status. Med Mycol. 2021;51:624–33. 10.1093/mmy/myaa112 33443574

[14] Wheat LJ, Connolly-Stringfield PA, Baker RL, Curfman MF, Eads ME, Israel KS, et al. Disseminated histoplasmosis in the acquired immune deficiency syndrome: clinical findings, diagnosis and treatment, and review of the literature. Medicine (Baltimore). 1990;69(6):361–74. doi: 10.1097/00005792-199011000-00004 2233233

[15] Kennedy CC, Limper AH. Redefining the clinical spectrum of chronic pulmonary histoplasmosis: a retrospective case series of 46 patients. Medicine (Baltimore). 2007;86(4):252–8. doi: 10.1097/MD.0b013e318144b1d9 17632267

[16] Goodwin RA Jr, Owens FT, Snell JD, Hubbard WW, Buchanan RD, Terry RT, et al. Chronic pulmonary histoplasmosis. Medicine (Baltimore). 1976;55(6):413–52. doi: 10.1097/00005792-197611000-00001 792626

[17] Baker J, Kosmidis C, Rozaliyani A, Wahyuningsih R, Denning DW. Chronic pulmonary histoplasmosis—a scoping literature review. Open Forum Infect Dis. 2020;7(5):ofaa119. doi: 10.1093/ofid/ofaa119 32411810 PMC7210804

[18] Kauffman CA. Histoplasmosis: a clinical and laboratory update. Clin Microbiol Rev. 2007;20(1):115–32. doi: 10.1128/CMR.00027-06 17223625 PMC1797635

[19] Rubin H, Furcolow ML, Yates JL, Brasher CA. The course and prognosis of histoplasmosis. Am J Med. 1959;27:278–88. doi: 10.1016/0002-9343(59)90347-x 14439879

[20] Wheat LJ, Wass J, Norton J, Kohler RB, French ML. Cavitary histoplasmosis occurring during two large urban outbreaks. Analysis of clinical, epidemiologic, roentgenographic, and laboratory features. Medicine (Baltimore). 1984;63(4):201–9. doi: 10.1097/00005792-198407000-00002 6738342

[21] Dall Bello AG, Severo CB, Guazzelli LS, Oliveira FM, Hochhegger B, Severo LC. Histoplasmosis mimicking primary lung cancer or pulmonary metastases. J Bras Pneumol. 2013;39(1):63–8. 23503487 10.1590/S1806-37132013000100009 PMC4075798

[22] Goodwin RA Jr, Owens FT, Snell JD, Hubbard WW, Buchanan RD, Terry RT, et al. Chronic pulmonary histoplasmosis. Medicine (Baltimore). 1976;55(6):413–52. doi: 10.1097/00005792-197611000-00001 792626

[23] Gurney JW, Conces DJ. Pulmonary histoplasmosis. Radiology. 1996;199(2):297–306. doi: 10.1148/radiology.199.2.8668768 8668768

[24] Chong S, Lee KS, Yi CA, Chung MJ, Kim TS, Han J. Pulmonary fungal infection: imaging findings in immunocompetent and immunocompromised patients. Eur J Radiol. 2006;59(3):371–83. doi: 10.1016/j.ejrad.2006.04.017 16725293

[25] Tobón AM, Gómez BL. Pulmonary histoplasmosis. Mycopathologia. 2021;186(5):697–705. 34498137 10.1007/s11046-021-00588-4

[26] Bankier AA, MacMahon H, Colby T, Gevenois PA, Goo JM, Leung ANC, et al. Fleischner society: glossary of terms for thoracic imaging. Radiology. 2024;310(2):e232558. 10.1148/radiol.232558 PMC10902601 38411514

[27] Goodwin RA Jr, Snell JD, Hubbard WW, Terry RT. Early chronic pulmonary histoplasmosis. Am Rev Respir Dis. 1966;93(1):47–61. 5901386 10.1164/arrd.1966.93.1.47

[28] Loewen DF, Procknow JJ, Loosli CG. Chronic active pulmonary histoplasmosis with cavitation. A clinical and laboratory study of thirteen cases. Am J Med. 1960;28:252–80. doi: 10.1016/0002-9343(60)90189-3 14417915

[29] Sutliff WD, Hughes F, Ulrich E, Burkett LL. Active chronic pulmonary histoplasmosis. AMA Arch Intern Med. 1953;92(4):571–86. doi: 10.1001/archinte.1953.00240220119015 13091474

[30] Parker JD, Sarosi GA, Doto IL, Bailey RE, Tosh FE. Treatment of chronic pulmonary histoplasmosis. N Engl J Med. 1970;283(5):225–9. 5424731 10.1056/NEJM197007302830503

[31] Unis G, Severo LC. Chronic pulmonary histoplasmosis mimicking tuberculosis. J Bras Pneumol. 2005;31(4):318–24.

[32] Pan B, Chen M, Pan W, Liao W. Histoplasmosis: a new endemic fungal infection in China? Review and analysis of cases. Mycoses. 2013;56(3):212–21. 23216676 10.1111/myc.12029

[33] Sousa C, Marchiori E, Youssef A, Mohammed TL, Patel P, Irion K. J Fungi (Basel). 2022;8(11). 10.3390/jof8111132 PMC9692403 36354899

[34] Hage CA, Knox KS, Wheat LJ. Endemic mycoses: overlooked causes of community acquired pneumonia. Respir Med. 2012;106(6):769–76. doi: 10.1016/j.rmed.2012.02.004 22386326

[35] Semionov A, Rossi A, Perillo M, Sayegh K, Pressacco J, Kosiuk J. Many faces of thoracic histoplasmosis – pictorial essay. Can Assoc Radiol J. 2019;70(3):273–81. 31104862 10.1016/j.carj.2018.12.006

[36] Goodwin RA Jr, Snell JD, Hubbard WW, Terry RT. Early chronic pulmonary histoplasmosis. Am Rev Respir Dis. 1966;93(1):47–61. doi: 10.1164/arrd.1966.93.1.47 5901386

[37] McKinsey DS, Pappas PG. Histoplasmosis: time to redraw the map and up our game. Clin Infect Dis. 2020;70(6):1011–3. doi: 10.1093/cid/ciz327 31038169

[38] Nacher M, Adenis A, Abboud P, Djossou F, Demar M, Epelboin L, et al. HIV patients dying on anti-tuberculosis treatment: are undiagnosed infections still a problem in French Guiana?. BMC Res Notes. 2020;13(1):209. doi: 10.1186/s13104-020-05054-w 32276647 PMC7149834

[39] Bahr NC, Antinori S, Wheat LJ, Sarosi GA. Histoplasmosis infections worldwide: thinking outside of the Ohio River valley. Curr Trop Med Rep. 2015;2(2):70–80. doi: 10.1007/s40475-015-0044-0 26279969 PMC4535725

[40] Ashraf N, Kubat RC, Poplin V, Adenis AA, Denning DW, Wright L, et al. Re-drawing the maps for endemic mycoses. Mycopathologia. 2020;185:843–65. 32040709 10.1007/s11046-020-00431-2 PMC7416457

[41] Denning DW. Global incidence and mortality of severe fungal disease. Lancet Infect Dis. 2024;24:e428–38. 10.1016/S1473-3099(23)00692-8 38224705

[42] FURCOLOW ML, BRASHER CA. Chronic progressive (cavitary) histoplasmosis as a problem in tuberculosis sanatoriums. Am Rev Tuberc. 1956;73(5):609–19. doi: 10.1164/artpd.1956.73.5.609 13313949

[43] Gugnani HC, Denning DW. Infection of bats with *Histoplasma* species. Med Mycol. 2023;61(8):myad080. doi: 10.1093/mmy/myad080 37553137 PMC10802898

[44] Diaz JH. Environmental and wilderness-related risk factors for histoplasmosis: more than bats in caves. Wilderness Environ Med. 2018;29(4):531–40. 30266238 10.1016/j.wem.2018.06.008

[45] Denning DW, Cadranel J, Beigelman-Aubry C, Ader F, Chakrabarti A, Blot S, et al. Chronic pulmonary aspergillosis: rationale and clinical guidelines for diagnosis and management. Eur Respir J. 2016;47(1):45–68. doi: 10.1183/13993003.00583-2015 26699723

[46] Mithunage CT, Denning DW. Timing of recurrence after treatment of pulmonary TB. IJTLD Open. 2024;1(10):456–65. doi: 10.5588/ijtldopen.24.0222 39398436 PMC11467853

[47] García-Marrón M, García-García JM, Pajín-Collada M, Alvarez-Navascués F, Martínez-Muñiz MA, Sánchez-Antuña AA. Chronic pulmonary histoplasmosis diagnosed in a nonimmunosuppressed patient 10 years after returning from an endemic area. Arch Bronconeumol. 2008;44(10):567–70. 19006637

[48] Gazzoni FF, Severo LC, Marchiori E, Irion KL, Guimarães MD, Godoy MC, et al. Fungal diseases mimicking primary lung cancer: radiologic–pathologic correlation. Mycoses. 2014;57(4):197–208. doi: 10.1111/myc.12150 24147761

[49] Croft DR, Trapp J, Kernstine K, Kirchner P, Mullan B, Galvin J. FDG-PET imaging and the diagnosis of non-small cell lung cancer in a region of high histoplasmosis prevalence. Lung Cancer. 2002;36(3):297–301. 12009241 10.1016/s0169-5002(02)00023-5

[50] Rolston KV, Rodriguez S, Dholakia N, Whimbey E, Raad I. Pulmonary infections mimicking cancer: a retrospective, three-year review. Support Care Cancer. 1997;5(2):90–3. doi: 10.1007/BF01262563 9069606

[51] Toscanini MA, Nusblat AD, Cuestas ML. Diagnosis of histoplasmosis: current status and perspectives. Appl Microbiol Biotechnol. 2021;105(5):1837–59. doi: 10.1007/s00253-021-11170-9 33587157

[52] Kauffman CA. Diagnosis of histoplasmosis in immunosuppressed patients. Curr Opin Infect Dis. 2008;21(4):421–5. doi: 10.1097/QCO.0b013e328306eb8d 18594296

[53] Azar MM, Hage CA. Laboratory diagnostics for histoplasmosis. J Clin Microbiol. 2017;55(6):1612–20. doi: 10.1128/JCM.02430-16 28275076 PMC5442517

[54] Global Action for Fungal Infections (GAFFI). Diagnostics for Fungal Disease in Africa: A GAFFI Survey. 2022 [cited 24 February 2025]. Available from: https://gaffi.org/africa-diagnostic-reports-2/.

[55] Swartzentruber S, Rhodes L, Kurkjian K, Zahn M, Brandt ME, Connolly P, et al. Diagnosis of acute pulmonary histoplasmosis by antigen detection. Clin Infect Dis. 2009;49(12):1878–82. doi: 10.1086/648421 19911965

[56] Hage CA, Ribes JA, Wengenack NL, Baddour LM, Assi M, McKinsey DS, et al. A multicenter evaluation of tests for diagnosis of histoplasmosis. Clin Infect Dis. 2011;53(5):448–54. 21810734 10.1093/cid/cir435

[57] Hage CA, Davis TE, Fuller D, Egan L, Witt JR 3rd, Wheat LJ, et al. Diagnosis of histoplasmosis by antigen detection in BAL fluid. Chest. 2010;137(3):623–8. doi: 10.1378/chest.09-1702 19837826

[58] Merino-Alado R, Alvarado P, Caceres D, Bastidas K, Mata-Essayag S, Salazar F, et al. Urinary antigen detection for the diagnosis of pulmonary histoplasmosis in a Venezuelan population. Poster presented at: ISHAM Congress; May 2025; Cataratas do Iguaçu, Brazil.

[59] Hage CA, Wheat LJ. Diagnosis of pulmonary histoplasmosis using antigen detection in the bronchoalveolar lavage. Expert Rev Respir Med. 2010;4(4):427–9. doi: 10.1586/ers.10.36 20658902

[60] Hage CA, Azar MM, Bahr N, Loyd J, Wheat LJ. Histoplasmosis: up-to-date evidence-based approach to diagnosis and management. Semin Respir Crit Care Med. 2015;36(5):729–45. doi: 10.1055/s-0035-1562899 26398539

[61] Muñoz C, Gómez BL, Tobón A, Arango K, Restrepo A, Correa MM, et al. Validation and clinical application of a molecular method for identification of *Histoplasma capsulatum* in human specimens in Colombia, South America. Clin Vaccine Immunol. 2010;17(1):62–7. doi: 10.1128/CVI.00332-09 19940044 PMC2812102

[62] Ohno H, Tanabe K, Umeyama T, Kaneko Y, Yamagoe S, Miyazaki Y. Application of nested PCR for diagnosis of histoplasmosis. J Infect Chemother. 2013;19(5):999–1003. doi: 10.1007/s10156-013-0548-2 23345048

[63] Carreto-Binaghi LE, Morales-Villarreal FR, García-de la Torre G, Vite-Garín T, Ramirez J-A, Aliouat E-M, et al. *Histoplasma capsulatum* and *Pneumocystis jirovecii* coinfection in hospitalized HIV and non-HIV patients from a tertiary care hospital in Mexico. Int J Infect Dis. 2019;86:65–72. doi: 10.1016/j.ijid.2019.06.010 31207386

[64] Ekeng BE, Oladele RO, Emanghe UE, Ochang EA, Mirabeau TY. Prevalence of histoplasmosis and molecular characterization of *Histoplasma* species in patients with presumptive pulmonary tuberculosis in Calabar, Nigeria. Open Forum Infect Dis. 2022;9(8):ofac368. doi: 10.1093/ofid/ofac368 36004316 PMC9397383

[65] Alanio A, Gits-Muselli M, Lanternier F, Sturny-Leclère A, Benazra M, Hamane S, et al. Evaluation of a new *Histoplasma* spp. quantitative RT-PCR assay. J Mol Diagn. 2021;23(6):698–709. doi: 10.1016/j.jmoldx.2021.02.007 33706012

[66] Davies SF. Serodiagnosis of histoplasmosis. Semin Respir Infect. 1986;1(1):9–15. 3317601

[67] LIFE Worldwide. [cited 15 November 2024]. Available from: http://en.fungaleducation.org/antibody-testing/.

[68] Salmanton-García J, Au W-Y, Hoenigl M, Chai LYA, Badali H, Basher A, et al. The current state of laboratory mycology in Asia/Pacific: a survey from the European Confederation of Medical Mycology (ECMM) and International Society for Human and Animal Mycology (ISHAM). Int J Antimicrob Agents. 2023;61(3):106718. doi: 10.1016/j.ijantimicag.2023.106718 36640851

[69] Almeida M d e A, Pizzini CV, Damasceno LS, Muniz M d e M, Almeida-Paes R, Peralta RH, et al. Validation of western blot for *Histoplasma capsulatum* antibody detection assay. BMC Infect Dis. 2016;16:87. 26905567 10.1186/s12879-016-1427-0 PMC4765212

[70] Straub M, Schwarz J. The healed primary complex in histoplasmosis. Am J Clin Pathol. 1955;25(7):727–41. doi: 10.1093/ajcp/25.7.727 14387973

[71] Armstrong PA, Beard JD, Bonilla L, Arboleda N, Lindsley MD, Chae SR, et al. Outbreak of severe histoplasmosis among tunnel workers – Dominican Republic, 2015. Clin Infect Dis. 2018;66(10):1550–7. 29211836 10.1093/cid/cix1067 PMC11034975

[72] Ketai L, Currie BJ, Holt MR, Chan ED. Radiology of chronic cavitary infections. J Thorac Imaging. 2018;33(5):334–43. doi: 10.1097/RTI.0000000000000346 30048346

[73] SCHWARZ J, BAUM GL, STRAUB M. Cavitary histoplasmosis complicated by fungus ball. Am J Med. 1961;31:692–700. doi: 10.1016/0002-9343(61)90153-x 13909773

[74] Holt MR, Chan ED. Chronic cavitary infections other than tuberculosis: clinical aspects. J Thorac Imaging. 2018;33(5):322–33. doi: 10.1097/RTI.0000000000000345 30036298

[75] Saab SB, Ungaro R, Almond C. The role and results of surgery in the management of chronic pulmonary histoplasmosis. J Thorac Cardiovasc Surg. 1974;68(1):159–67. 4834077

[76] Furcolow ML, Doto IL. Course and prognosis of untreated histoplasmosis. A United States Public Health Service Cooperative Mycoses Study. JAMA. 1961;177:292–6. 14447643

[77] Kosmidis C, Newton P, Muldoon EG, Denning DW. Chronic fibrosing pulmonary aspergillosis: a cause of “destroyed lung” syndrome. Infect Dis (Lond). 2017;49(4):296–301. doi: 10.1080/23744235.2016.1232861 27658458

[78] Gadkowski LB, Stout JE. Cavitary pulmonary disease. Clin Microbiol Rev. 2008;21(2):305–33, table of contents. doi: 10.1128/CMR.00060-07 18400799 PMC2292573

[79] Libshitz HI, Atkinson GW, Israel HL. Pleural thickening as a manifestation of *Aspergillus* superinfection. Am J Roentgenol Radium Ther Nucl Med. 1974;120(4):883–6. doi: 10.2214/ajr.120.4.883 4821341

[80] Rumbak M, Kohler G, Eastrige C, Winer-Muram H, Gavant M. Topical treatment of life threatening haemoptysis from aspergillomas. Thorax. 1996;51(3):253–5. doi: 10.1136/thx.51.3.253 8779126 PMC1090634

[81] Soeroso NN, Siahaan L, Khairunnisa S, Anggriani RAH, Aida A, Eyanoer PC, et al. The association of chronic pulmonary aspergillosis and chronic pulmonary histoplasmosis with MDR-TB patients in Indonesia. J Fungi (Basel). 2024;10(8):529. doi: 10.3390/jof10080529 39194855 PMC11355089

[82] Muldoon EG, Sharman A, Page I, Bishop P, Denning DW. *Aspergillus* nodules; another presentation of chronic pulmonary aspergillosis. BMC Pulm Med. 2016;16(1):123. doi: 10.1186/s12890-016-0276-3 27538521 PMC4991006

[83] de Oliveira CV, Horvat N, Testagrossa LA, Romão DDS, Rassi MB, Lee HJ. Etiological profile and main imaging findings in patients with granulomatous diseases who underwent lung biopsy. Eur J Radiol Open. 2021;8:100325. 33521170 10.1016/j.ejro.2021.100325 PMC7820493

[84] de Matos PMPG, Felipe-Silva A, Otoch JP. Pulmonary histoplasmoma: a disguised malady. Autops Case Rep. 2018;8(4):e2018065. doi: 10.4322/acr.2018.065 30775333 PMC6360832

[85] Baum GL, Bernstein IL, Schwarz J. Broncholithiasis produced by histoplasmosis. Am Rev Tuberc. 1958;77(1):162–7. doi: 10.1164/artpd.1958.77.1.162 13498302

[86] Seo JB, Song K-S, Lee JS, Goo JM, Kim HY, Song J-W, et al. Broncholithiasis: review of the causes with radiologic–pathologic correlation. Radiographics. 2002;22 Spec No:S199-213. doi: 10.1148/radiographics.22.suppl_1.g02oc07s199 12376611

[87] Rossi SE, McAdams HP, Rosado-de-Christenson ML, Franks TJ, Galvin JR. Fibrosing mediastinitis. Radiographics. 2001;21(3):737–57. doi: 10.1148/radiographics.21.3.g01ma17737 11353121

[88] Lanzillotta M, Culver E, Sharma A, Zen Y, Zhang W, Stone JH, et al. Fibrotic phenotype of IgG4-related disease. Lancet Rheumatol. 2024;6(7):e469–80. doi: 10.1016/S2665-9913(23)00299-0 38574746

[89] Mazi PB, Arnold SR, Baddley JW, Bahr NC, Beekmann SE, McCarty TP, et al. Management of histoplasmosis by infectious disease physicians. Open Forum Infect Dis. 2022;9(7):ofac313. doi: 10.1093/ofid/ofac313 35899286 PMC9310261

[90] Furcolow ML. Comparison of treated and untreated severe histoplasmosis: a communicable disease center cooperative mycoses study. JAMA. 1963;183(10):823–9.

[91] Bongomin F, Asio LG, Baluku JB, Kwizera R, Denning DW. Chronic pulmonary aspergillosis: notes for a clinician in a resource-limited setting where there is no mycologist. J Fungi (Basel). 2020;6(2). 10.3390/jof6020075 PMC7345130 32498415

[92] Lewis R, Niazi-Ali S, McIvor A, Kanj SS, Maertens J, Bassetti M, et al. Triazole antifungal drug interactions-practical considerations for excellent prescribing. J Antimicrob Chemother. 2024;79(6):1203–17. doi: 10.1093/jac/dkae103 38629250 PMC11977760

[93] Lewis R, Niazi-Ali S, McIvor A, Sharah SK, Maertens J, Bassetti M, et al. Triazole antifungal drug interactions – practical considerations for excellent prescribing. J Antimicrob Chemother. 2024;79:1203–17. 38629250 10.1093/jac/dkae103 PMC11977760

[94] Wheat J, Sarosi G, McKinsey D, Hamill R, Bradsher R, Johnson P, et al. Practice guidelines for the management of patients with histoplasmosis. Clin Infect Dis. 2000;30(4):688–95. 10770731 10.1086/313752

[95] Varghese C, Johnson GB, Eiken PW, Edell ES, Specks U, Larson NB, et al. A retrospective evaluation of the treatment effects of rituximab in patients with progressive and symptomatic fibrosing mediastinitis. Ann Am Thorac Soc. 2024;21(11):1533–41. doi: 10.1513/AnnalsATS.202405-533OC 39106522 PMC12392372

